# Predictors of long-term mortality in older patients with hip fractures managed by hemiarthroplasty: a 10-year study based on a population registry in Saxony, Germany

**DOI:** 10.1186/s13037-024-00398-9

**Published:** 2024-04-30

**Authors:** Johannes K.M. Fakler, Philipp Pieroh, Andreas Höch, Andreas Roth, Christian Kleber, Markus Löffler, Christoph E. Heyde, Samira Zeynalova

**Affiliations:** 1 Department of Orthopaedic, Trauma and Plastic Surgery, University Hospital of Leipzig, Liebigstr. 20, 04103 Leipzig, Germany; 2 Department of Orthopaedic and Trauma Surgery, Hospital of Passau, Innstr. 76, 94032 Passau, Germany; 3 Institute for Medical Informatics, Statistics and Epidemiology (IMISE), University of Leipzig, Härtelstrasse 16-18, 04107 Leipzig, Germany

**Keywords:** Femoral neck fracture, Hip fracture, Long-term survival, Mortality

## Abstract

**Background:**

Mortality of patients with a femoral neck fracture is high, especially within the first year after surgery, but also remains elevated thereafter. The aim of this study was to identify factors potentially associated with long-term mortality in patients homogeneously treated with hemiarthroplasty for femoral neck fracture.

**Methods:**

This retrospective cohort study was performed at a single level 1 national trauma center at the university hospital of Leipzig (Saxony, Germany). The study time-window was January 1, 2010 to December 31, 2020. Primary outcome measure was mortality depending on individual patient-related characteristics and perioperative risk factors. Inclusion criteria was a low-energy femoral neck fracture (Garden I-IV) in geriatric patients 60 years of age or older that were primarily treated with bipolar hemiarthroplasty. Date of death or actual residence of patients alive was obtained from the population register of the eastern German state of Saxony, Germany. The outcome was tested using the log-rank test and plotted using Kaplan-Meier curves. Unadjusted and adjusted for other risk factors such as sex and age, hazard ratios were calculated using Cox proportional hazards models and presented with 95% confidence intervals (CI).

**Results:**

The 458 included patients had a median age of 83 (IQR 77–89) years, 346 (75%) were female and 113 (25%) male patients. Mortality rates after 30 days, 1, 5 and 10 years were 13%, 25%, 60% and 80%, respectively. Multivariate regression analysis revealed age (HR = 1.1; *p* < 0.001), male gender (HR = 1.6; *p* < 0.001), ASA-Score 3–4 vs. 1–2 (HR = 1.3; *p* < 0.001), dementia (HR = 1.9; *p* < 0.001) and a history of malignancy (HR = 1.6; *p* = 0.002) as independent predictors for a higher long-term mortality risk. Perioperative factors such as preoperative waiting time, early surgical complications, or experience of the surgeon were not associated with a higher overall mortality.

**Conclusions:**

In the present study based on data from the population registry from Saxony, Germany the 10-year mortality of older patients above 60 years of age managed with hemiarthroplasty for femoral neck fracture was 80%. Independent risk factors for increased long-term mortality were higher patient age, male gender, severe comorbidity, a history of cancer and in particular dementia. Perioperative factors did not affect long-term mortality.

## Background

Due to demographic changes the number of geriatric fractures is constantly increasing [1]. Within the last 10 years hip fractures increased by more than 20% in Germany [1]. Compared to the general elderly population, geriatric patients with a hip fracture are exposed to excess mortality [2–4]. Excess mortality is highest within the first months after injury and decreases over time but remains elevated in mid- to long-term (5 to 10 years) follow-up studies [2–4].

Short-term relative mortality risk after hip fracture surgery may be associated with peri- and postoperative events, such as pulmonary embolism [5], infectious complications [6, 7], or heart failure [8, 9]. Additionally, general health status and accompanying comorbid conditions, such as dementia, cardiovascular disease, or a history of malignancy increase short-term mortality [10–12]. Specific laboratory parameters were also linked to short-term mortality [13, 14]. However, much less information is available on predictable characteristics regarding long-term survival of 5 years and beyond [15–17].

This study aimed to analyze long-term mortality and potentially associated factors in patients with a femoral neck fracture homogenously treated with hemiarthoplasty.

## Methods

### Study design

This retrospective cohort study was performed at a single level 1 national trauma center at the university hospital of Leipzig (Saxony, Germany). The study time-window was January 1, 2010 to December 31, 2020. Primary outcome measure was mortality depending on individual patient-related characteristics and perioperative risk factors. Inclusion criteria was a low-energy femoral neck fracture (Garden I-IV) in geriatric patients 60 years of age or older that were primarily treated with bipolar hemiarthroplasty. Exclusion criteria was severely multiple injuries (polytrauma), pathologic fracture due to metastatic bone disease and previous fracture of the contralateral hip. Furthermore, patients in which date of death or actual residency could not be identified were excluded.

Baseline and treatment data of all patients were retrieved from the electronic patient charts. The following data were documented: age, gender, body mass index (BMI), ASA-Score [18], diagnosis of medical diseases, medication, routine laboratory parameters as well as peri- and intraoperative data, time to surgery and experience of the surgeon (resident vs. senior surgeon). Time to surgery or preoperative waiting time was defined as time from admission to skin incision. Over the study period a total of 29 orthopaedic resident and senior surgeons performed one or more hemiarthoplasties. Furthermore, early postoperative surgical complications (e.g. hematoma/seroma, surgical site infection, periprosthetic fractures and hip dislocation) were recorded. To identify periprosthetic and trochanteric fractures all intra- and postoperative x-rays were screened.

Information regarding the date of death or actual residence of patients alive was obtained from the population register of the eastern German state of Saxony, Germany in early 2022.

Data processing and analysis was performed in accordance with national data protection regulations.

The study was approved by the local Ethics Committee at the University of Leipzig 2021-04-13 (ref. 144/21-ek). The procedures used in this study adhere to the tenets of the Declaration of Helsinki.

### Patient selection

**Fig. 1:**
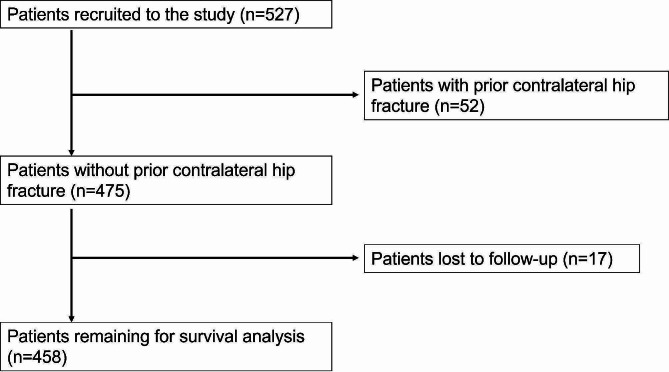
Flow-Chart demonstrating patient selection of the study population

A total of 527 patients with a minimum age of 60 years patients and a low-energy femoral neck fracture treated with a bipolar hemiarthroplasty were identified in the study period. Of these 527 patients 52 were excluded due to a previous fracture of the contralateral hip and 17 patients were excluded, because an actual residence or a date of death could not be determined. Consequently, 458 patients remained for survival analysis [Fig. 1].

### Surgical treatment

Surgery was performed in general anesthesia in all patients. Patients were placed in a supine position. A modified lateral (Hardinge) or anterolateral approach was performed in all patients.

A bipolar hemiprosthesis (DePuy Synthes, USA) was the standard implant at our institution. Cemented stems were used in 91% of patients and uncemented stems in 9% patients. All patients were allowed to fully weight bearing and were instructed to use a walker or two crutches, if possible.

### Statistics

Survival analysis was performed with the log-rank test plotted with Kaplan-Meier curves. The Cox proportional hazards model was constructed to calculate the hazard ratio (HR) and the associated 95% confidence interval (CI) to evaluate the increased risk of mortality associated with patient specific and perioperative risk factors. Unadjusted and adjusted HRs of mortality were computed. A statistically significant difference was considered when *p* ≤ 0.05. Statistical analyses were performed using SPSS statistics version 26.

## Results

Median age of all patients was 83 (IQR 77–89) years, 345 (75%) were female and 113 (25%) male patients, median BMI was 24 (IQR 22–27). The majority of patients presented with severe comorbidities according to the ASA classification grade 3 (71%), followed by grade 2 (25%), grade 4 (2%) and grade 1 (1%). With respect to fracture classification 92% were graded as displaced (Garden 3 and 4) and 8% non-displaced (2% Garden I, 6% Garden II). Surgical treatment was initiated within 24 h after admission in 49% of all patients and in 51% beyond 24 h after admission. Postoperative surgical complications were recorded in 61 (13%) patients: 29 greater trochanteric fractures, 17 early surgical site infections, 12 hematomas/seromas, 3 dislocations. Revision surgery was necessary in 27 (6%) patients.

**Fig. 2:**
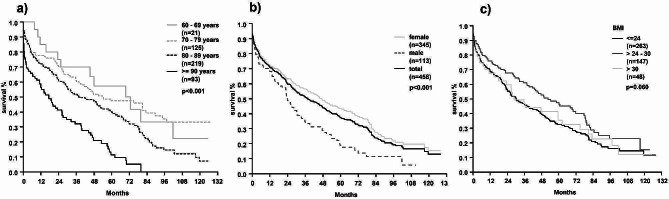
Kaplan-Meier curves comparing mortality rates between **a**) age groups, **b**) gender and **c**) BMI categories

Cumulated mortality rates after 30 days, 1, 5 and 10 years were 13%, 25%, 60% and 80%, respectively. The mortality rates by age group for patients younger than 70 years, those aged 71–80 years, those aged 81–90 years, and those aged 91 years and older were 0%, 9%, 9%, and 24% at 30 days postoperatively; 15%, 22%, 28%, and 44% at one-year; 43%, 52%, 60%, and 88% at five-years [Fig. 2a].

Female patients presented with significantly lower long-term mortality rates than male patients [Fig. 2b]. The difference of mortality rates between genders increased over time. The 30-day mortality was 11% for females vs. 14% for males, at 1 and 5 years 28% vs. 32% and 57% vs. 81%, respectively. Yearly consecutive mortality was most pronounced in women within the first postoperative year and in men within the first three postoperative years [Table 1].

In univariate analysis, long-term survival was better in patients with a BMI between 25 and 30 (normal to moderate overweight) compared to patients with normal weight, underweight or obese, but the difference was not significant. [Fig. 2c].

**Fig. 3:**
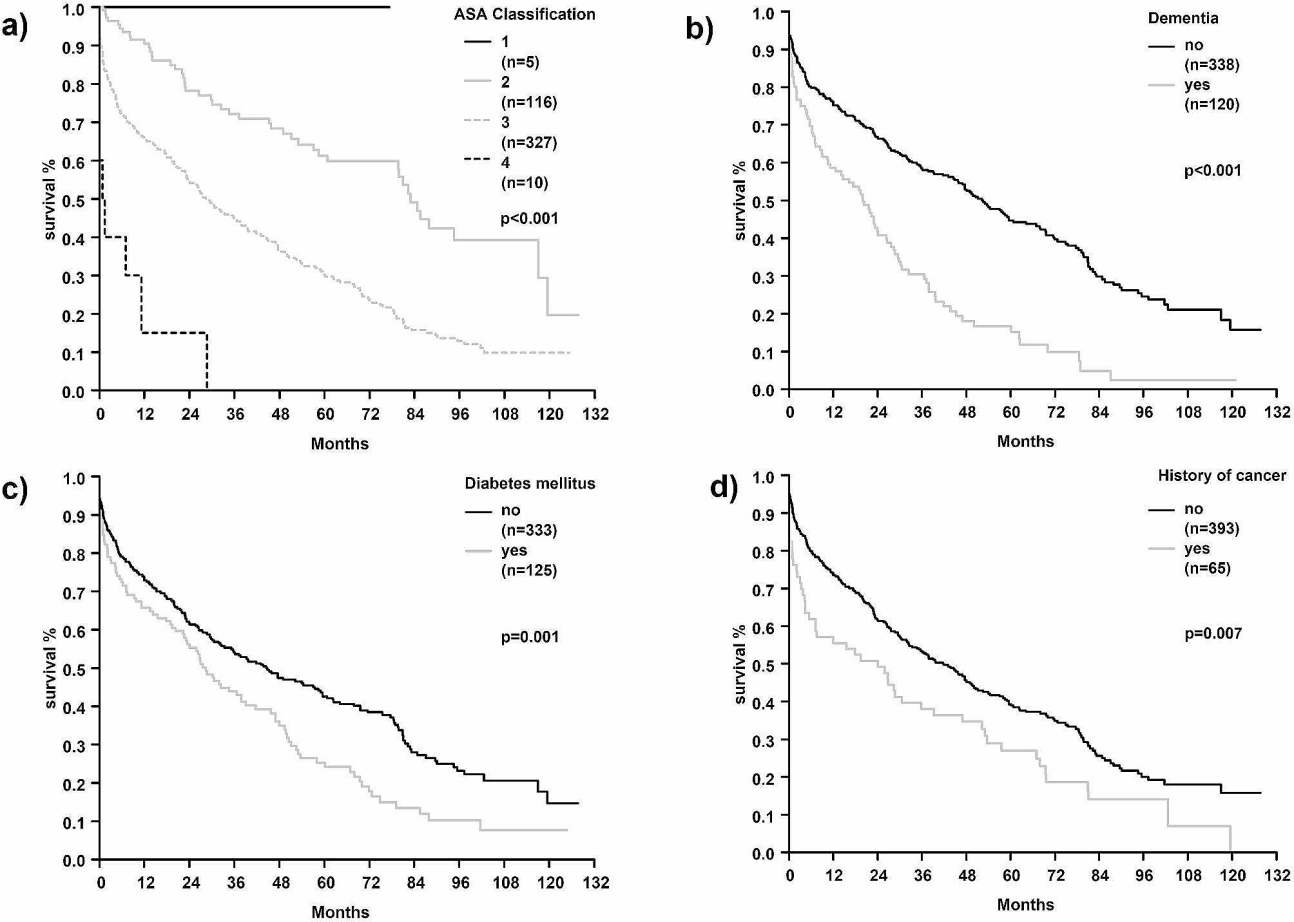
Kaplan-Meier curves comparing mortality rates between **a**) ASA class, **b**) patient with and without dementia, **c**) patients with and without diabetes mellitus and **d**) patients with and without a history of cancer

ASA classification, dementia, diabetes and a history of cancer was significantly associated with increased post-operative long-term mortality [Fig. 3a].

In patients with dementia the mortality rate at 30 days was 17% and in patients without dementia 10%. Mortality increased over time to 41% at one year in patients with dementia compared to 25% in patients without dementia and to 83% vs. 55% at five years, respectively [Fig. 3b].

In patients with diabetes, short-term mortality at 30 days was 15% compared with 10% in patients without diabetes and 34% vs. 27% at one year increasing to 75% vs. 57% at five years [Fig. 3c].

Long-term mortality was also significantly different in patients with a history of cancer compared to those without (*p* = 0.007). Mortality rates at 30 days, 1 and 5 years were 22% vs. 10%, 44% vs. 26% and 73% vs. 61%, respectively [Fig. 3d].

**Fig. 4:**
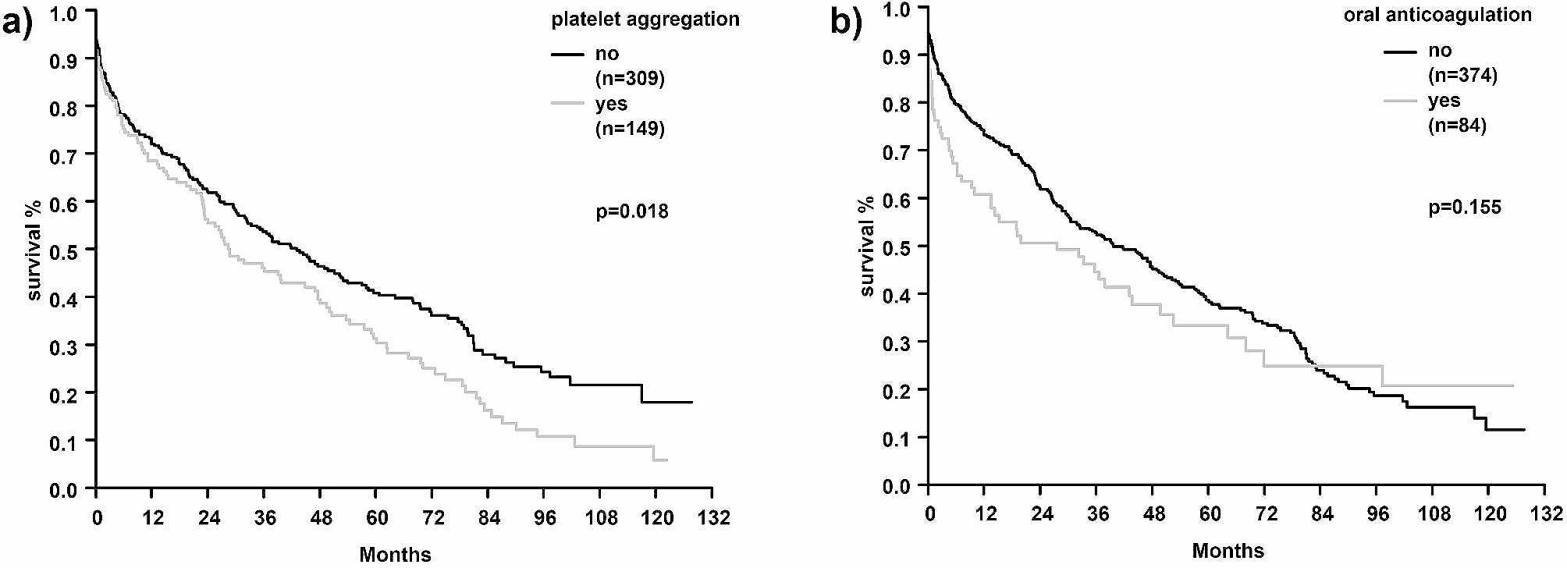
Kaplan-Meier curves comparing mortality rates between patients (a) with and without platelet inhibitor medication and (b) with and without oral anticoagulation

Patients treated with a platelet inhibitor displayed a significantly higher long-term mortality rate compared to non-platelet-inhibitor treated patients [Fig. 4a]. The treatment with anticoagulants did not affect long-term mortality [Fig. 4b].

**Fig. 5:**
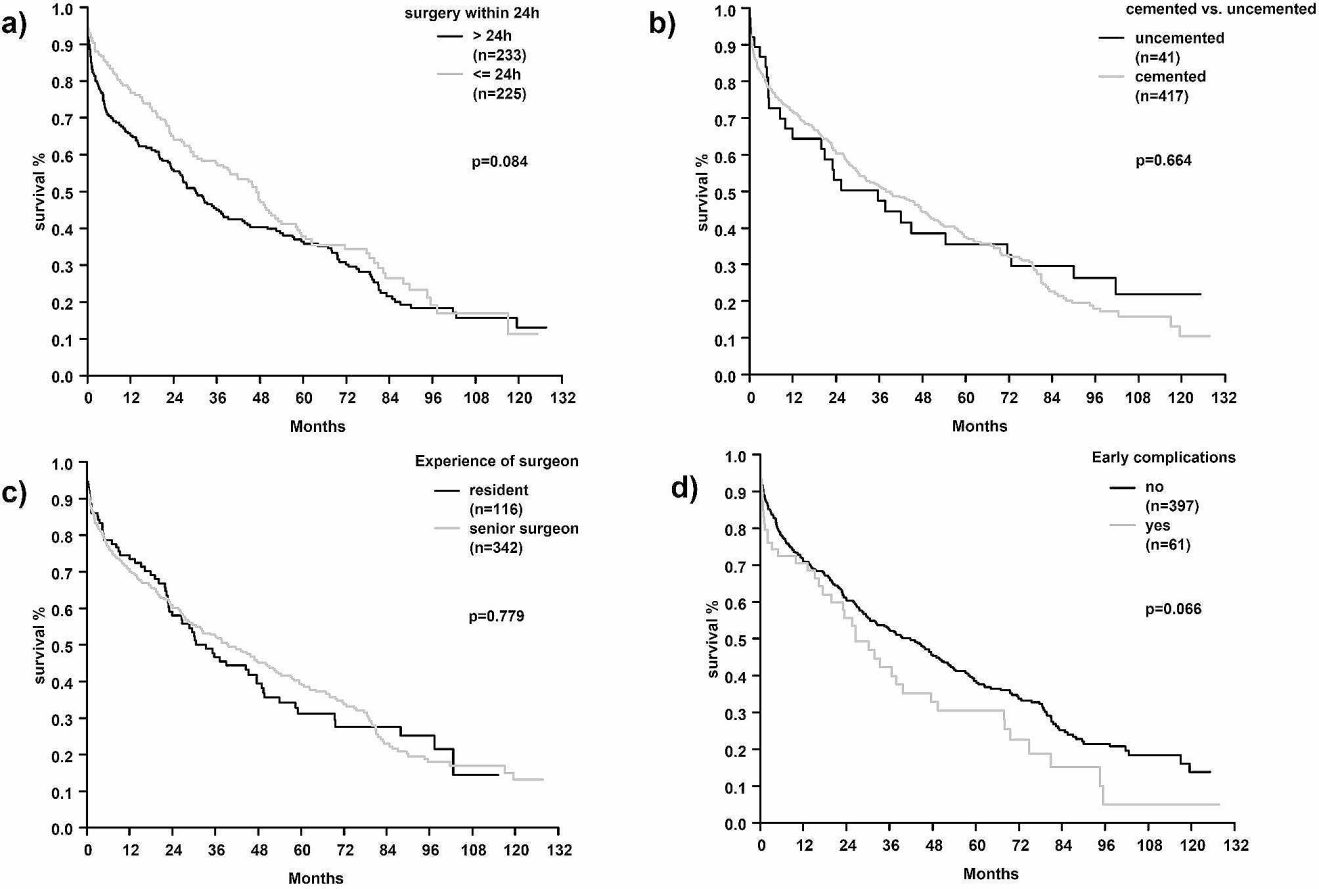
Kaplan-Meier curves comparing mortality rates between patients **a**) having surgery within or beyond 24 h after admission, **b**) receiving a cemented or uncemented stem, **c**) treated by a resident or senior surgeon d) with or without experiencing early complications within 3 months postoperatively

Peri- and intraoperative factors, such as preoperative waiting time [Fig. 5a], choice of stem fixation (cemented vs. uncemented) [Fig. 5b], duration of surgery, surgery on call and experience of the surgeon [Fig. 5c] did not influence long-term mortality rates. Postoperative surgical complications also did not significantly impair long-term survival, although the difference spread over time [Fig. 5d].

Independent factors associated with mortality were identified with multivariate analysis. Age (HR = 1.1; *p* < 0.001), male gender (HR = 1.6; *p* < 0.001), ASA-Score > 2 (3–4 vs. 1–2, HR = 1.3; *p* < 0.001), dementia (HR = 1.9; *p* < 0.001) and a history of malignancy (HR = 1.6; *p* = 0.002) were found to be independent predictors for a higher long-term mortality risk. In contrary, BMI (HR = 1.0; *p* = 0.153) and diabetes mellitus (HR = 1.3; *p* = 0.065) did not reach a level of significance [Table 2].

## Discussion

Treatment of intracapsular femoral neck fractures in geriatric patients with hemiarthoplasty is a generally accepted procedure [19]. In contrary, limited value is attributed to hemiarthoplasty due to superior functional results and higher long-term implant survival of THA [20]. However, our study revealed that over two thirds of patients had severe comorbidity questioning a high functional demand. Moreover, in light of a 10-year mortality rate of 80% long-term survival of implants may be of secondary interest. Nevertheless, identification of long-term mortality predictors in this context may help to differentiate which patients may profit from THA or hemiarthoplasty. Valgus-impacted femoral neck fractures (Graden type I) may also be a potential treatment option [21]. But in our study cohort, this fracture type is only represented by 2% of patients and to our knowledge no long-term mortality data are available for conservatively managed femoral neck fractures.

In terms of mortality hemiarthoplasty seems to be comparable to total hip arthroplasty (THA), despite of several differences as for example higher blood loss and longer duration of surgery associated with THA on the one hand and better functional results for THA on the other hand [20, 22, 23]. Similarly, mortality rates after internal fixation (IF) and hemiarthoplasty in an elderly patient cohort exhibit no significant differences [24, 25]. Moreover, intra- and extraarticular hip fractures demonstrate similar mortality rates, regardless of surgical treatment modality [26, 27]. Thus, it seems legitimate to compare our study cohort, which exclusively included femoral neck fractures treated with bipolar hemiarthoplasty, with other hip fracture cohorts irrespective of specific hip fracture type or surgical treatment.

However, few studies report on differences in mortality with reference to IF compared to arthroplasty in femoral neck fractures [28–30]. As discussed earlier [30], these differences may especially be ascribed to selection bias. Lin and Liang [31] decreased, but did not eliminate, selection bias by matching a cohort of 13,772 patients with non-displaced femoral neck fracture treated with IF to a equivalent cohort in size with non-displaced femoral neck fractures treated with hemiarthroplasty according to age, gender and level of Charlson Mobility Index (CCI). Mortality rates were 13.4% and 14.6% after 1 year, 43.7% and 46.9% after 5 years, 67.1% and 71% after 10 years, respectively. Regarding the whole observation period of 10 years, the log rank test demonstrated a significant benefit for IF. From a clinical point of perspective, a mortality difference of 0.9% after one year and 3.9% after 10 years may be less striking or even be interpreted as rather comparable. Nevertheless, these mortality rates are substantially lower than in our study with 1-, 5- and 10-year mortality rates of 25%, 60% and 80% which indeed must be regarded as clinically relevant. But this difference can be explained by much older patients included in our study with a median age of 83 (IQR 77–89) compared to the afore mentioned study [31] with a mean age of 76 ± 8 years. In addition, mortality rates (78% at 1 and 48% at 5years) of our subgroup of patients aged 70–79 years (median age 75) compared favorably to the study of Lin and Liang [31]. Our patient cohort is also somewhat older compared to other studies [16, 17, 27, 32], because we included only patients that were treated with hemiarthroplasty which is preferentially considered for older hip fracture patients. However, short- and long-term mortality rates were comparable to others [16, 17, 27, 32].

Miettinen et al. [16] followed 241 operatively treated hip fracture patients for up to 10 years [16]. Mean age of all patients was 81.4 (SD 6.8) years and 76% of patients were female. They reported cumulative mortality rates of 4% and 14% after 30 days, 23% vs. 28% after 1 year, 63% vs. 75% after 5 years and 87% vs. 88% after 10 years for females and males, respectively. We found mortality rates for females and males of 11% vs. 14% at 30 days, 28% vs. 32% at 1 year, 57% vs. 81% at 5 years. Interestingly, Mettinen et al. [16] found no difference of mortality between both genders which is in opposition to our results and that of others [17, 27, 33]. Differences in mortality rates between both genders became most apparent at approximately 5 years after surgery, which was almost one third lower in females. Similar mortality rates with 57% for females and 73% for males at 5 years were also exhibited by Knauf et al. [27]. Moreover, Paksima et al. [17] identified male patients aged 65 to 84 years having the highest mortality risk.

Compared with the general population mortality rates in our study are considerable higher, especially in the first years. Mortality rates of elderly women aged 80 to 85 years in the general population range from 4 to 7% [34] compared with a female mortality rate of 28% in the first year after surgery decreasing to 8% in the second postoperative year in our study. The mortality rate of elderly men aged 80 to 85 years in the general population is 6–10% [34]. In our male study cohort mortality rates persisted at high rates within the first 3 years after surgery and then declined. This is in accordance to a meta-analysis by Haentjes et al. [4] that reported a pronounced excess mortality within the first two years after hip fracture surgery and declining thereafter, but persisting over the follow-up period of 10 years.

Apart from age and gender, comorbidity significantly influences mortality risk in hip fracture patients [16, 17, 27]. Similarly, multivariable analysis exhibited the ASA-Score as an independent predictor of mortality in our study. Patients with diagnosed dementia revealed the most abrupt increase in mortality with 16%, 36%, 55% and 82% after 30 days, 1, 2 and 5 years. Schaller et al. [35] reported a one year mortality of 18% for patients with mild to moderate dementia which was a more than 5 fold increase compared to those without cognitive impairment. Interestingly, 1-year mortality in those patients was twice as high than in our study. An explanation might be that we included only patients with diagnosed dementia and missed several patients with milder to moderate forms. In opposition, Schaller et al. [35] excluded patients with a Mini-Mental State Examination (MMSE) less than 15. A lower MMSE was also found to be an independent risk factor for death up to 5 years after hip fracture surgery by others [27].

Even Xue et al. detected a significantly higher 1-year mortality and risk of death for hip fracture patients with dementia, comparable to our results [36]. Similar 30 day and 1-year mortality rates compared to our study were also confirmed in a study that included 9394 hip fracture patients with dementia. Overall mortality was 13% and 37% after 30 days and 1 year, respectively [37]. Of note, it was demonstrated that arthroplasty was protective over internal fixation in terms of 1-year mortality in this subgroup of hip fracture patients [36, 37]. With regard to the short life expectancy of cognitive impaired patients demonstrating a 5-year survival rate of less than 20% in our study hemiarthroplasty seems to be a valid treatment option.

Apart from dementia, diabetes mellitus, a history of malignant disease and use of platelet inhibitors as a surrogate for cardiovascular disease were associated with an increased mortality in our study which is also supported by others [17, 38]. As demonstrated by others [39], oral anticoagulants had no effect on long-term mortality in our study.

Preoperative waiting time over 24 h was associated with a higher mortality in univariate analysis. After correcting for age, gender and comorbidities in a multivariate analysis a prolonged waiting time was no longer a risk factor of mortality. It could be hypothesized that patients with comorbidities may require more time for preoperative conditioning, consequently exposing preoperative waiting time to bias. However, the influence of time-to-surgery is still discussed controversially which may in part depend on different national health systems and different variables included in national registries [40–44]. We also found no influence of the surgeons´ experience on mortality rates which is also reported by others [16, 45]. Revision surgery for early surgical complications was necessary in 6% of our study cohort which is similar to other studies [32, 45]. Overall early surgical complications occurring in 13% of our study cohort affected mortality throughout the observation period, but did not reach a level of significance. This might be explained by a relatively high rate of trochanteric fractures accounting for 47% of surgical complications. Trochanteric fractures result in loss of hip abductor strength and hip stability with consecutive impaired mobility [46] which could in turn have an effect on mortality.

This study is limited by its retrospective and single-center design. In addition, patients were not screened for cognitive impairment and only patients with diagnosed dementia were respected in this subgroup. Consequently, it must be assumed that patients with a mild to moderate form of dementia were missed for this subgroup analysis. Although a considerable amount of confounding parameters were considered in multivariate analysis, unmeasured or unknown confounders were not entirely respected which is inherent to all retrospective studies.

The strength of this study lies in the integrity of officially reported date of death which could be reviewed for every included patient with no loss of follow-up. Second, a focus on hip fracture patients exclusively treated with hemiarthroplasty with no change of perioperative and anesthesiologic procedures as well as continuous use of the same implant manufacturer over more than a decade potentially reduced bias. Third, combining short- and long-term observation may aid in differentiating between surgery and patient individual effects on mortality.

## Conclusions

In summary, the 10-year mortality of elder patients above 60 years of age managed with hemiarthroplasty for femoral neck fracture was 80%. Independent risk factors for increased long-term mortality were higher patient age, male gender, severe comorbidity, a history of cancer and in particular dementia. Perioperative factors did not affect long-term mortality. Due to the drastically limited long-term survival rate and an acceptable surgical revision rate, hemiarthoplasty may be considered as a safe and viable treatment option in elderly patients with a femoral neck fracture.

**Table 1:** Yearly consecutive mortality rate (%) after hemiarthoplasty for femoral neck fracture

year	1	2	3	4	5	6	7	8	9	10
**overall**	29.1	10.9	8.8	7.1	6.6	5.2	8.5	4.5	2.7	3.7
**women**	28.1	8.1	7.1	7.4	5.7	5.3	10.4	5.9	2.1	4.4
**men**	31.9	19.5	14.0	5.8	9.7	5.4	2.3	1.1	0.6	0.6

**Table 2:** Multivariate analysis of risk factors for long-term mortality

	P-Value	HR	95% CI
**age**	< 0.001	1.1	1.0–1.1
**gender (m)**	< 0.001	1.6	1.2–2.1
**BMI**	0.153	1.0	0.9–1.0
**ASA classifikation**	< 0.001	1.3	1.2–1.4
**diabetes mellitus**	0.065	1.3	1.0–1.6
**malignancy in history**	0.002	1.6	1.2–2.3
**dementia**	< 0.001	1.9	1.5–2.5

## Data Availability

No datasets were generated or analysed during the current study.
